# New thresholds in semi-quantitative [^18^F]FDG PET/CT are needed to assess large vessel vasculitis with long-axial field-of-view scanners

**DOI:** 10.1007/s00259-023-06423-w

**Published:** 2023-09-07

**Authors:** Luisa Knappe, Carola Bregenzer, Nasir Gözlügöl, Clemens Mingels, Ian Alberts, Axel Rominger, Federico Caobelli

**Affiliations:** Department of Nuclear Medicine, Inselspital, University Hospital Bern, University of Bern, Freiburgstrasse 18, 3011 Bern, Switzerland

**Keywords:** Large vessel vasculitis, [^18^F]FDG PET, Long-axial field-of-view PET, Total-body PET, Inflammation

## Abstract

**Aim:**

[^18^F]FDG PET/CT proved accurate in the diagnostic work-up of large vessel vasculitis (LVV). While a visual interpretation is currently considered adequate, several attempts have been made to integrate it with a semiquantitative evaluation. In this regard, there is the need to validate current or new thresholds for the semiquantitative parameters on long-axial field of view (LAFOV) scanners.

**Methods:**

We retrospectively evaluated 100 patients (50 with LVV and 50 controls) who underwent [^18^F]FDG LAFOV PET/CT. Semiquantitative parameters (SUVmax and SUVmean) were calculated for large vessels in 3 districts (supra-aortic [SA], thoracic aorta [TA], and infra-aortic [IA]). Values were also normalized to liver activity (SUV_max_/L-SUV_max_, and SUV_max_/L-SUV_mean_).

**Results:**

Of the 50 patients diagnosed with LVV, SA vessels were affected in 38 (76%), TA in 42 (84%) and IA vessels in 26 (52%). To-liver normalized values had higher diagnostic accuracy than non-normalized values (AUC always ≥ 0.90 vs. 0.74–0.89). For the SA vessels, best thresholds were 0.66 for SUV_max_/L-SUV_max_ and 0.88 for SUV_max_/L-SUV_mean_; for the TA, 1.0 for SUV_max_/L-SUV_max_ and 1.30 for SUV_max_/L-SUV_mean_; finally, for IA vessels, the best threshold was 0.83 for SUV_max_/L-SUV_max_ and 1.11 for SUV_max_/L-SUV_mean_.

**Conclusion:**

LAFOV [^18^F]FDG-PET/CT is accurate in the diagnostic workup of LVV, but different threshold in semi-quantitative parameters than reported in literature for standard scanners should be considered.

**Supplementary Information:**

The online version contains supplementary material available at 10.1007/s00259-023-06423-w.

## Introduction

^18^F-2-Fluoro-2-deoxy-d-glucose ([^18^F]FDG) positron emission tomography/computed tomography (PET/CT) has secured an important role in the diagnosis and follow-up of large vessel vasculitis (LVV) [1–3]. Current guidelines recommend a visual evaluation of [^18^F]FDG PET with a standardized grading system based on the comparison with liver uptake, wherein uptake in a vessel equal to that of the liver is rated as possibly positive (grade 2) and uptake greater than that in the liver as definitively positive (grade 3) [4].

Although visual interpretation is robust if readers are expert, semi-quantitative values have been suggested, which may help in the diagnostic work-up by increasing readers’ confidence. In two recent publications, the ratio between maximum standardized uptake value (SUV_max_) within a large vessel and the mean SUV in the liver (L-SUV_mean_) has been suggested as the most accurate semi-quantitative parameter for the diagnosis of LVV. Specifically, a ratio of 1.0 for supra-aortic (SA) vessels and 1.3 for thoracic aorta (TA) and infra-aortic (IA) vessels yielded the best diagnostic performance [5, 6].

However, validated semi-quantitative values and their thresholds rely on studies featuring analogue PET scanners only. It is well known that semi-quantitative parameters, especially SUV_max_ can vary considerably across different scanners [7, 8]. This appears of utmost importance for studies performed on newer generation digital scanners, including long-axial field-of-view (LAFOV) PET/CT systems. The limited resolution and poor signal recovery of small structures such as the vessel wall are partially overcome by digital systems using silicon photomultiplier (SiPM) systems [9]. Such scanners have improved spatial and temporal resolution compared to analogue systems [8, 10] and impact thresholds in the semiquantitative evaluation in the assessment of LVV.

As such, we aimed to evaluate the diagnostic accuracy of various semi-quantitative parameters in [^18^F]FDG LAFOV PET/CT and to identify the most accurate thresholds.

## Materials and methods

### Patient population

We retrospectively evaluated the first 50 patients with a final clinical diagnosis of LVV who had undergone [^18^F]FDG PET/CT for the diagnostic work-up of LVV starting from November 2020. All patients were referred for evaluation of a new diagnosis of LVV. No patients were included who underwent therapy assessment or follow up in known LVV. The final diagnosis of LVV was reached in a multidisciplinary setting based on laboratory results, clinical symptoms and imaging results (ultrasound and PET). All patients were scanned on a LAFOV PET/CT system (Biograph Vision Quadra, Siemens Healthineers, Erlangen, Germany) within 48 h from the first clinical consult, wherein LVV was suspected. As control group, another 50 patients were randomly selected from our oncologic database. To that end, all patients were free from signs of vasculitis. Patients with lymphomas, those referred for the search for infectious or inflammatory foci and those patients under therapy with monoclonal antibodies were excluded.

### Imaging protocol

Patients fasted for at least 6 h prior to scanning, blood glucose levels were always  < 120 mg/dl (6.7 mmol/L). Sixty minutes after the intravenous administration of a weight-adapted activity of [^18^F]FDG (3.0 MBq/kg), images were acquired on the LAFOV PET/CT scanner in list-mode for 10 min in a single bed position (skull-vertex to mid femur). Image reconstruction was performed as previously described using high sensitivity mode (HS, maximum ring difference of 85) [10].

Whole body PET images were reconstructed in 3D to a 440 × 440 × 644 matrix with a voxel size of 1.65 × 1.65 × 1.65 mm^3^, with a zoom factor of 1.0 using the proprietary time of flight (TOF) point-spread-function (PSF) algorithm with 4 iterations and 5 subsets. A Gauss filter was applied (2-mm FWHM). Emission data were corrected for randoms, scatter, and decay. Non-contrast enhanced, low-dose CT images were used for attenuation correction, parameters have been also previously published [8].

### Image evaluation

Images were visually and semi-quantitatively evaluated using appropriate workstation (Syngo.via MMOncology, Siemens Healthineers, Erlangen, Germany). Semi-quantitative parameters (SUV_max_, SUV_mean_, and SUV_peak_, respectively) were calculated in the relevant large vessels by manually placing a volume-of-interest (VOI) with a 40%-iso-contour around their whole diameter. Large vessels were divided into three groups: (1) SA vessels: temporal, carotid and subclavian arteries; (2) TA; and (3) IA vessels: abdominal aorta, external/internal iliac, and femoral arteries. Values were calculated for each group, as mean of the single values of all the relevant large vessels.

Consistent with previous reports [5], to-liver normalized values were also calculated for all vessels as the ratio of SUV_max_ of the relevant vessel to SUV_max_ of the liver (L-SUV_max_) and SUV_mean_ of the liver (L-SUV_mean_). To that end, these semi-quantitative parameters were calculated for the liver also by placing a standard 10 cm^3^ VOI in healthy liver tissue in the right lobe. To-blood pool (BP) normalized values were also calculated, by placing a standard 2 × 2 pixels wide VOI centred at the mitral valve plane.

### Statistical analysis

Statistical analysis was carried out using IBM SPSS (Version 28.0.1.1, IBM Corp. Armonk, NY, USA). Data are presented as mean ± SD. Comparison between continuous variables in patients with and without LVV was tested using Mann–Whitney *U* test. The diagnostic accuracy of different semi-quantitative parameters was evaluated by means of receiver-operating-characteristic (ROC) curves analysis on a per-patient basis (with 95% CI) with calculation of Youden Index for the assessment of the best thresholds. *P* values < 0.05 were considered statistically significant.

## Results

### Semi-quantitative parameters

Of the 50 patients diagnosed with LVV, SA vessels were affected in 38 (76%), thoracic aorta in 42 (84%), and IA vessels in 26 (52%). Clinical charachteristics are displayed in Table 1. Only 3 patients with LVV (6.0%) presented with the involvement of only one segment (2 with involved temporal arteries and 1 with left subclavian artery), while the majority of patients had multilevel involvement. All semi-quantitative parameters were significantly different between patients with and without LVV (Table 2). Of note, L-SUV_max_ was also different between patients with and without LVV (2.98 ± 0.13 vs. 3.32 ± 0.14, *p* = 0.027), while L-SUV_mean_ was not (2.51 ± 0.10 vs. 2.32 ± 0.09, *p* = 0.125).

**Table 1:** Clinical characteristics of the patients with LVV

Clinical characteristic	*n* (%)
Amaurosis fugax	3 (6.0%)
Loss of vision	2 (4.0%)
New onset headache	24 (48.0%)
Jaw claudication	1 (2.0%)
Scalp tenderness	10 (20.0%)
Pathological temporal artery	7 (14.0%)
Proximal muscle pain	19 (38.0%)
Fever	8 (16.0%)
Erythrocyte sedimentation rate	62 mm/h (16–101)
C-reactive-protein	24.3 mg/L (10–61)

**Table 2:** Age and semi-quantitative values in patients with and without large vessel vasculitis (LVV). Data are provided as mean ± SD. Area under the curve (AUC) is provided with 95% confidence intervals (CI)

	SA vessels vasculitis				TA vasculitis				IA vessels vasculitis			
	Pos (*n* = 38)	Neg (*n* = 62)	*p*	AUC (95% CI)	Pos (*n* = 42)	Neg (*n* = 58)	*p*	AUC (95% CI)	Pos (*n* = 26)	Neg (*n* = 74)	*p*	AUC (95% CI)
Age (years)	69.3 ± 9.34	66.5 ± 11.7	0.283	-	68.5 ± 9.97	66.9 ± 11.5	0.546	-	69.2 ± 8.18	67.0 ± 11.7	0.563	-
Male gender, *n* (%)	13 (34.2)	28 (45.2)	0.301	-	13 (31.0)	28 (48.3)	0.101	-	8 (30.8)	33 (44.6)	0.253	-
SUVmax	3.48 ± 1.53	1.89 ± 0.57	< 0.01	0.81 (0.73–0.90)	4.62 ± 1.69	2.50 ± 0.97	< 0.01	0.88 (0.81–0.95)	4.02 ± 1.41	2.95 ± 1.47	< 0.01	0.89 (0.83–0.96)
SUVmean	1.92 ± 0.67	1.28 ± 0.39	< 0.01	0.76 (0.67–0.86)	2.12 ± 0.61	1.60 ± 0.62	< 0.01	0.78 (0.69–0.87)	2.04 ± 0.62	1.73 ± 0.66	0.012	0.79 (0.70–0.88)
SUVpeak	2.32 ± 0.87	1.57 ± 0.45	< 0.01	0.74 (0.64–0.84)	3.19 ± 0.96	2.06 ± 0.73	< 0.01	0.85 (0.77–0.93)	2.69 ± 0.82	2.26 ± 0.92	0.008	0.84 (0.77–0.92)
SUVmax/L-SUVmax	1.22 ± 0.61	0.60 ± 0.13	< 0.01	0.91 (0.85–0.97)	1.62 ± 0.61	0.77 ± 0.19	< 0.01	0.95 (0.92–0.99)	1.45 ± 0.56	0.94 ± 0.45	< 0.01	0.98 (0.96–1.00)
SUVmax/L-SUVmean	1.61 ± 0.72	0.80 ± 0.19	< 0.01	0.90 (0.84–0.96)	2.07 ± 0.75	1.01 ± 0.26	< 0.01	0.94 (0.90–0.99)	1.88 ± 0.68	1.23 ± 0.56	< 0.01	0.98 (0.95–1.00)

*SUV* Standardized uptake value, *L-SUV* to-liver normalized standardized uptake value, *SA* supra-aortic vessels, *TA* thoracic aorta, *IA* infra-aortic vessels, *Pos.* patients with LVV, *Neg.* patients without LVV

### Per-patient diagnostic accuracy

**Fig. 1:**
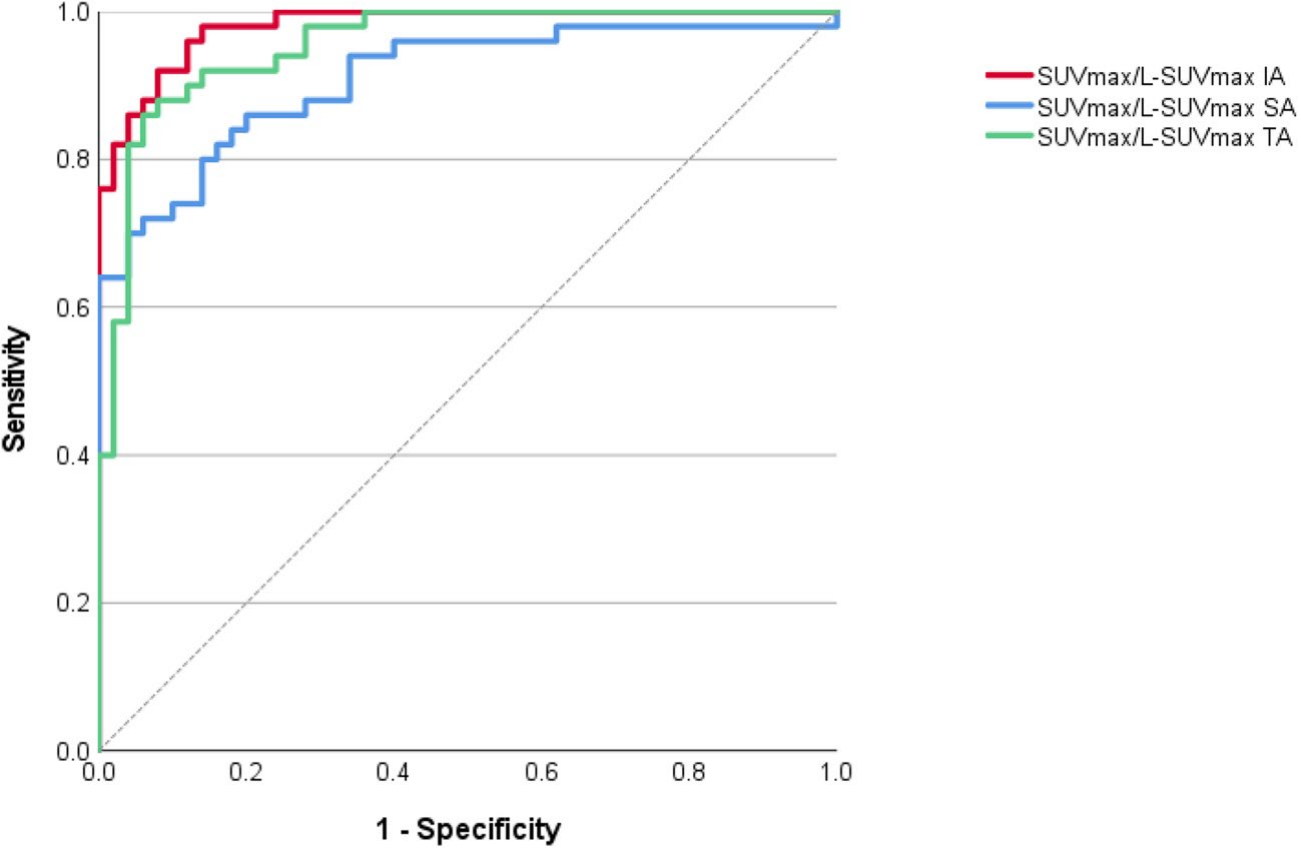
ROC-curves analysis for normalized maximum standardized uptake value (SUV_max_) of the relevant vessels to liver SUV_max_ (L-SUV_max_). SA = supra-aortic vessels; TA = thoracic aorta; IA = infra-aortic vessels

**Fig. 2:**
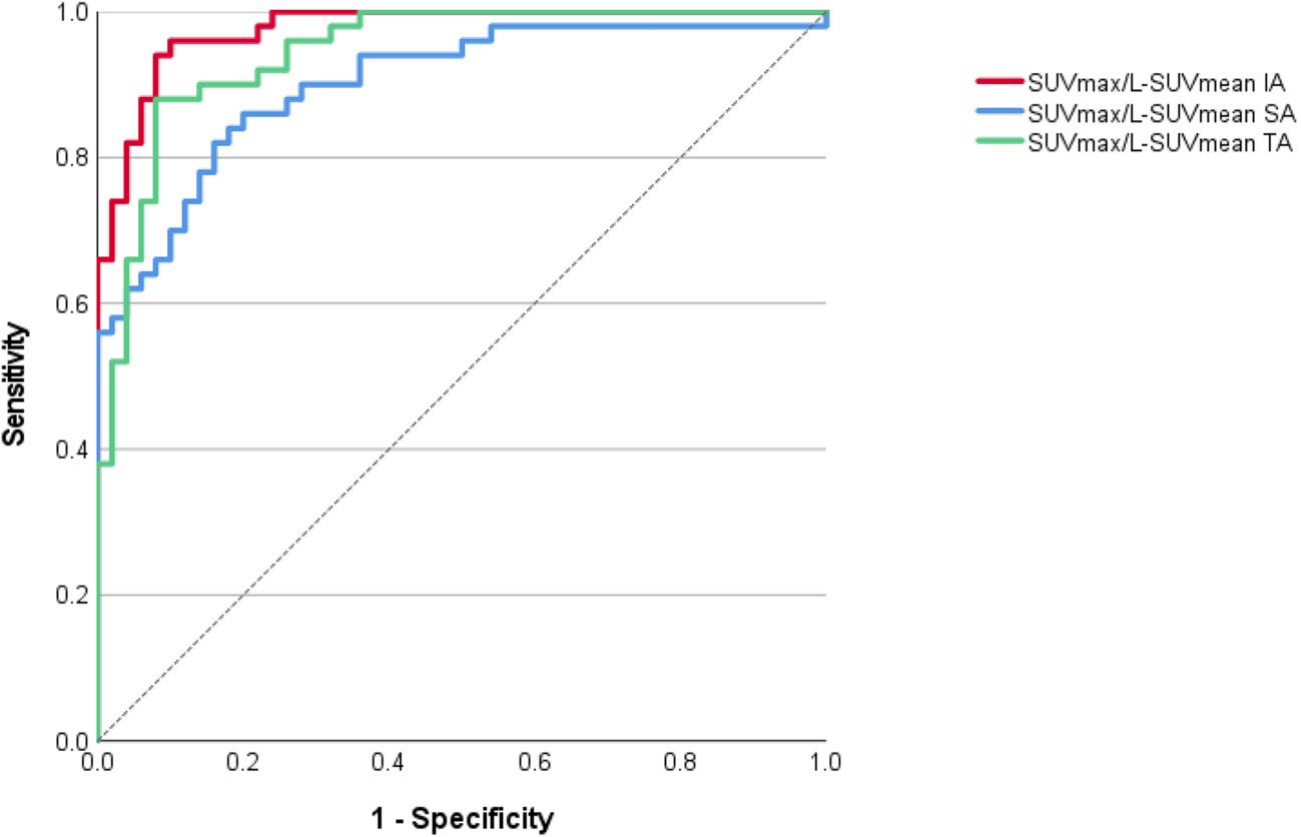
ROC-curves analysis for normalized maximum standardized uptake value (SUV_max_) of the relevant vessels to liver SUVmean (L-SUV_mean_). SA = supra-aortic vessels; TA = thoracic aorta; IA = infra-aortic vessels

ROC-curves analysis showed higher diagnostic accuracy for to-liver normalized values (AUC always ≥ 0.90) than for non-normalized ones (AUC 0.74–0.89, Table 2). Best separators were calculated for to-liver normalized values, whose ROC curves are displayed in Figs. 1 and 2. For the SA vessels, best thresholds were 0.66 for SUV_max_/L-SUV_max_ and 0.88 for SUV_max_/L-SUV_mean_; for the TA, 1.0 for SUV_max_/L-SUV_max_ and 1.30 for SUV_max_/L-SUV_mean_; finally, for IA vessels, the best threshold was 0.83 for SUV_max_/L-SUV_max_ and 1.11 for SUV_max_/L-SUV_mean_. The thresholds and their sensitivity and specificity are reported in Table 3. To-blood pool normalized ratio proved less accurate (Supplemental Table).

**Table 3:** Best separators for semi-quantitative values with values of sensitivity and specificity

	Threshold	Sensitivity	Specificity
SUVmax SA/L-SUVmax	0.66	86%	80%
SUVmax AT/L-SUVmax	1.00	88%	92%
SUVmax IA/L-SUVmax	0.83	98%	86%
SUVmax SA/L-SUVmean	0.88	86%	80%
SUVmax AT/L-SUVmean	1.30	88%	92%
SUVmax IA/ L-SUVmean	1.11	96%	90%

*SUV* Standardized uptake value, *L-SUV* to-liver normalized standardized uptake value, *SA* supra-aortic vessels, *TA* thoracic aorta, *IA* infra-aortic vessels

### Accuracy of to-date suggested thresholds on LAFOV PET

The currently suggested thresholds showed inferior sensitivity and slight higher specificity when applied to LAFOV PET imaging. Using the current threshold of SUV_max_/L-SUV_mean_ = 1.0 for SA vessels, sensitivity was 68% and specificity 90%, (with threshold 0.88 sensitivity was 86% and specificity 80%). Using the threshold of SUV_max_/L-SUV_mean_ = 1.3 for IA vessels resulted in sensitivity 80% and specificity 96% for IA vessels (with threshold 1.11, sensitivity 96% and specificity 90%).

## Discussion

While current guidelines recommend visual interpretation only in PET imaging in the assessment of LVV, recent evidence suggests that semi-quantitative methods may be preferred in clinical practice. Besides the abovementioned report on semi-quantitative parameters able to assist the clinicians in the diagnosis of LVV [5, 6], other scoring systems have been recently suggested [11], also able to differentiate between LVV and atherosclerosis with very good accuracy.

In this regard, it should be noted that a meta-analysis showed that the pooled sensitivity of a visual interpretation is good but not excellent, ranging between 75.9 and 83.3% using the clinical diagnosis as reference standard [12]. Adding also uptake intensity to the visual analysis yields higher diagnostic accuracy, as demonstrated by a recent prospective study featuring 64 patients with suspected giant cell arteritis, wherein PET had sensitivity 92% and specificity 85% [13].

As such, there is a clear rationale to pursue a semi-quantitative evaluation in PET imaging interpretation, although to-date there is still insufficient evidence of a superiority over visual interpretation only [3, 14]. However, another two questions arise: (1) what is the best semi-quantitative parameter and with which separator? and (2) is there a chance that semi-quantitative thresholds are not interchangeable across different PET scanners?

Our work expands on this topic, confirming that the to-liver normalized semi-quantitative values yield higher accuracy than non-normalized ones, consistent with previous reports [5, 6]. But in contrast to the previous works, we here demonstrate a substantial equivalence between SUV_max_/L-SUV_max_ and SUV_max_/L-SUV_mean_ in all vascular territories. Furthermore, we provide for the first time data on the diagnostic accuracy of semi-quantitative PET using LAFOV scanners.

We found that the best separators are lower than reported for conventional, analogue PET systems. The most conceivable explanation relies on the intrinsic differences in the scanners. On LAFOV PET, using a 10-min acquisition 1 h post injection reduces the background noise compared to a standard-axial field-of-view scanner (SAFOV) [15]. Moreover, using the advantage of covering all coincidences in the whole FOV simultaneously with a LAFOV system leads to a gain in scanner sensitivity. It should be noted that the exact contribution from digital vs. analogue systems rather than LAFOF vs. SAFOV could not be elucidated.

Thresholds reported in the literature should be decreased and updated when scanning patients on LAFOV PET systems, in order to avoid false negative findings. It should be acknowledged that the to-date established SUV_max_/L-SUV_mean_ threshold of 1.3 is adequate for inflammation involving the thoracic aorta. We highly recommend using normalized values as we showed to avoid inter-individual variations of [^18^F]FDG-uptake.

Taken together, our results seem to indicate a complementary role of the semi-quantitative PET analysis. As a matter of fact, a global scan assessment by PET-experienced nuclear medicine physicians including both visual and semi-quantitative analysis provides high accuracy [13], and this may reflect the fact that an experienced reader can weigh the impact of the degree of [^18^F]FDG uptake depending on the location, the pattern of [^18^F]FDG-uptake (i.e., diffuse vs. focal) and the presence of possible atheroma [13].

Some limitations of our study should be acknowledged. First, due to the retrospective nature of the present study, patients already diagnosed with LVV were evaluated, and their results were compared with patients without any clinical and radiologic sign of LVV. Hence, conclusions about our proposed thresholds in patients with clinical signs of LVV but unclear diagnosis may not be fully applicable in clinical practice. To note, the same limitation also pertains to previous studies featuring analogue PET scanners [5, 6]. Furthermore, the patients’ sample is relatively small and further prospective studies are needed for their precise definition. However, the main aim of our study was to underline the need for different thresholds when using LAFOV PET, which bears importance in clinical practice for the nuclear medicine community.

It should also be noted that we cannot rule out incorrect information on ongoing steroid therapy in our population. This may have impacted our results, as glucocorticoids may lower [^18^F]FDG uptake and mask a subtending inflammation. In this regard, a recent study showed a decrease in the [^18^F]FDG uptake after 3 days of high-dose glucocorticoids, but without loss of diagnostic accuracy, which only occurred after 10 days of treatment [16]. In clinical practice, the start of glucocorticoid therapy cannot often be delayed during severe clinical symptoms, and therefore, it is conceivable that some of our patients were on steroid therapy at the time of PET/CT. But given the fact that PET was always performed within 48 h from the first clinical visit, it is extremely unlikely that patients were on medications for more than 3 days. Hence, the impact on our values is expected to be negligible.

The multisciplinary team was aware of the PET/CT results as they provided relevant information for patient management, and this may have an impact on the diagnosis. However, the team has ample clinical experience, and based its judgment on the extensive information available from all sources at the end of the diagnostic work-up. Although this gold standard may be subject to criticism, still it represents a common clinical situation, wherein biopsy cannot be performed, and is adhering to current recommendations [17]. Finally, we could not assess the impact of a different timing of imaging after [^18^F]FDG injection. While different timing was reported to affect the sensitivity for active vasculitis, being higher for later acquisitions [18], our study features the same uptake period post injection of the previous reports, wherein the current thresholds for semi-quantitative PET were suggested. As such, there is a full comparability among our studies, which gives more reliability when assessing the need of different thresholds using LAFOV-PET.

## Conclusion

Our results confirm the importance of [^18^F]FDG-PET/CT in the diagnostic workup of LVV. In this regard, LAVOF PET/CT may yield increased diagnostic accuracy owing to reduced background noise and improved spatial/temporal resolution, but different thresholds in semi-quantitative parameters should be considered. Prospective studies are warranted to implement new reference values in clinical practice when using high sensitivity scanners.

### Supplementary Information

Below is the link to the electronic supplementary material.

Supplementary file1 (DOCX 12 KB)

## Data Availability

Data will be made available upon reasonable request.

## References

[1] Knappe LM, Verburg FA, Giovanella L, Luster M, Librizzi D (2023). Diagnostic value of FDG-PET/CT in the diagnostic work-up of inflammation of unknown origin. Nuklearmedizin.

[2] Palestro CJ, Brandon DC, Dibble EH, Keidar Z, Kwak JJ (2023). FDG PET in evaluation of patients with fever of unknown origin: AJR expert panel narrative review. AJR Am J Roentgenol.

[3] Slart RHJA, Nienhuis PH, Glaudemans AWJM, Brouwer E, Gheysens O, van der Geest KSM (2023). Role of 18F-FDG PET/CT in large vessel vasculitis and polymyalgia rheumatica. J Nucl Med.

[4] Slart RHJA; Writing group; Reviewer group; Members of EANM Cardiovascular; Members of EANM Infection & Inflammation; Members of Committees, SNMMI Cardiovascular; Members of Council, PET Interest Group; Members of ASNC; EANM Committee Coordinator. FDG-PET/CT(A) imaging in large vessel vasculitis and polymyalgia rheumatica: joint procedural recommendation of the EANM, SNMMI, and the PET Interest Group (PIG), and endorsed by the ASNC. Eur J Nucl Med Mol Imaging. 2018;45:1250–1269. 10.1007/s00259-018-3973-8 PMC5954002 29637252

[5] Imfeld S, Rottenburger C, Schegk E, Aschwanden M, Juengling F, Staub D, Recher M, Kyburz D, Berger CT, Daikeler T (2018). [18F]FDG positron emission tomography in patients presenting with suspicion of giant cell arteritis-lessons from a vasculitis clinic. Eur Heart J Cardiovasc Imaging.

[6] Imfeld S, Scherrer D, Mensch N, Aschwanden M, Staub D, Berger CT, Daikeler T, Rottenburger C (2022). A simplified PET/CT measurement routine with excellent diagnostic accuracy for the diagnosis of giant cell arteritis. Diagnostics (Basel).

[7] Dondi F, Pasinetti N, Gatta R, Albano D, Giubbini R, Bertagna F (2022). Comparison between two different scanners for the evaluation of the role of 18F-FDG PET/CT semiquantitative parameters and radiomics features in the prediction of final diagnosis of thyroid incidentalomas. J Clin Med.

[8] Mingels C, Weidner S, Sari H, Buesser D, Zeimpekis K, Shi K, Alberts I, Rominger A (2023). Impact of the new ultra-high sensitivity mode in a long axial field-of-view PET/CT. Ann Nucl Med.

[9] Alberts I, Prenosil G, Sachpekidis C, Weitzel T, Shi K, Rominger A, Afshar-Oromieh A (2020). Digital versus analogue PET in [^68^Ga]Ga-PSMA-11 PET/CT for recurrent prostate cancer: a matched-pair comparison. Eur J Nucl Med Mol Imaging.

[10] Alberts I, Sari H, Mingels C, Afshar-Oromieh A, Pyka T, Shi K, Rominger A (2023). Long-axial field-of-view PET/CT: perspectives and review of a revolutionary development in nuclear medicine based on clinical experience in over 7000 patients. Cancer Imaging.

[11] Bacour YAA, van Kanten MP, Smit F, Comans EFI, Akarriou M, de Vet HCW, Voskuyl AE, van der Laken CJ, Smulders YM. Development of a simple standardized scoring system for assessing large vessel vasculitis by 18F-FDG PET-CT and differentiation from atherosclerosis. Eur J Nucl Med Mol Imaging. 2023;50:2647–55. 10.1007/s00259-023-06220-5 PMC10317865 37115211

[12] Lee YH, Choi SJ, Ji JD, Song GG (2016). Diagnostic accuracy of 18F-FDG PET or PET/CT for large vessel vasculitis : a meta-analysis. Z Rheumatol.

[13] Sammel AM, Hsiao E, Schembri G, Nguyen K, Brewer J, Schrieber L, Janssen B, Youssef P, Fraser CL, Bailey E, Bailey DL, Roach P, Laurent R (2019). Diagnostic accuracy of positron emission tomography/computed tomography of the head, neck, and chest for giant cell arteritis: a prospective, double-blind, cross-sectional study. Arthritis Rheumatol.

[14] Gheysens O, Jamar F, Glaudemans AWJM, Yildiz H, van der Geest KSM (2021). Semi-quantitative and quantitative [18F]FDG-PET/CT indices for diagnosing large vessel vasculitis: a critical review. Diagnostics (Basel).

[15] Alberts I, Hünermund JN, Prenosil G, Mingels C, Bohn KP, Viscione M, Sari H, Vollnberg B, Shi K, Afshar-Oromieh A, Rominger A (2021). Clinical performance of long axial field of view PET/CT: a head-to-head intra-individual comparison of the Biograph Vision Quadra with the Biograph Vision PET/CT. Eur J Nucl Med Mol Imaging.

[16] Nielsen BD, Gormsen LC, Hansen IT, Keller KK, Therkildsen P, Hauge EM (2018). Three days of high-dose glucocorticoid treatment attenuates large-vessel 18F-FDG uptake in large-vessel giant cell arteritis but with a limited impact on diagnostic accuracy. Eur J Nucl Med Mol Imaging.

[17] Hellmich B, Sanchez-Alamo B, Schirmer JH, Berti A, Blockmans D, Cid MC, Holle JU, Hollinger N, Karadag O, Kronbichler A, Little MA, Luqmani RA, Mahr A, Merkel PA, Mohammad AJ, Monti S, Mukhtyar CB, Musial J, Price-Kuehne F, Segelmark M, Teng YKO, Terrier B, Tomasson G, Vaglio A, Vassilopoulos D, Verhoeven P, Jayne D. EULAR recommendations for the management of ANCA-associated vasculitis: 2022 update. Ann Rheum Dis. 2023:ard-2022–223764. 10.1136/ard-2022-223764 36927642

[18] Quinn KA, Rosenblum JS, Rimland CA, Gribbons KB, Ahlman MA, Grayson PC (2020). Imaging acquisition technique influences interpretation of positron emission tomography vascular activity in large-vessel vasculitis. Semin Arthritis Rheum.

