# Supplementary material for: New thresholds in semi-quantitative [18F]FDG PET/CT are needed to assess large vessel vasculitis with long-axial field-of-view scanners
Source: Eur J Nucl Med Mol Imaging. 2023 Sep 7;50(13):3890–6. doi: 10.1007/s00259-023-06423-w (PMC10611821; doi:10.1007/s00259-023-06423-w)
Supplement: Supplementary file 1 — Supplementary file1 (DOCX 12 KB) [file 259_2023_6423_MOESM1_ESM.docx]

| **Semiquantitative value** | **AUC** |
| --- | --- |
| SUVmax SA/BP-SUVmax | 0.907 |
| SUVmean SA/BP-SUVmean | 0.847 |
| SUVpeak SA/BP-SUVpeak | 0.868 |
| SUVmax TA/BP-SUVmax | 0.874 |
| SUVmean TA/BP-SUVmean | 0.866 |
| SUVpeak TA/BP-SUVpeak | 0.860 |
| SUVmax IA/BP-SUVmax | 0.825 |
| SUVmean IA/BP-SUVmean | 0.818 |
| SUVpeak IA/BP-SUVpeak | 0.786 |

Supplemental Table: to- blood pool (BP) normalized value with corresponding AUC
